# A ratiometric near‐infrared fluorescent probe for the detection and monitoring of hypochlorous acid in rheumatoid arthritis model and real water samples

**DOI:** 10.1002/smo.20220007

**Published:** 2023-03-21

**Authors:** Zhuye Shang, Xinyi Yang, Qingtao Meng, Shengye Tian, Zhiqiang Zhang

**Affiliations:** ^1^ School of Chemical Engineering University of Science and Technology Liaoning Anshan China; ^2^ Key Laboratory for Functional Material Educational Department of Liaoning Province University of Science and Technology Liaoning Anshan China

**Keywords:** bioimaging, fluorescence probe, hypochlorous acid, real water samples, rheumatoid arthritis

## Abstract

Recent studies revealed that the increased level of hypochlorous acid (HOCl) may be deemed to be one of the signs of chronic inflammatory joint disease. Accordingly, the development of effective methods for rapid and accurate detection or monitoring of HOCl in vivo is of great significance for further understanding the role of HOCl in rheumatoid arthritis (RA). Herein, a ratiometric near‐infrared (NIR) fluorescent probe (**PTA**) was reported for the detection and monitoring of HOCl. In the presence of HOCl, the electron‐rich S atom and C=C double bond of probe **PTA** were oxidized in sequence, resulting in the significant hypochromatic shift and decline of absorption spectra. Simultaneously, the intramolecular charge transfer (ICT) process of **PTA** is inhibited, causing the intrinsic fluorescence emission of **PTA** shift from 680 to 550 nm. **PTA**‐based test paper strips were successfully prepared and applied to determinate HOCl in actual water samples by “naked eye” colorimetric method. **PTA** features NIR emission, large Stokes shift (200 nm), low cytotoxicity, high sensitivity (33.9 nM), and short response time (45 s), which enable it to be successfully utilized for imaging endogenous and exogenous HOCl in living zebrafish and mice. More importantly, **PTA** shows remarkable effectiveness for the monitoring of HOCl‐mediated treatment response to RA. Consequently, **PTA** provides a new approach to further understand the role of HOCl in RA and evaluate the drug treatment efficiency of RA.

## Graphical abstract

A ratiometric near‐infrared (NIR) fluorescent probe (**PTA**) was reported for the detection and monitoring of HOCl. The electron‐rich S atom and C=C double bond of probe **PTA** were oxidized by HOCl in sequence, resulting in the significant hypochromatic shift of absorption and emission spectra. The **PTA**‐based test paper strips were successfully applied to determinate HOCl in real water samples by “naked eye” colorimetric method. Furthermore, **PTA** was successfully utilized for imaging endogenous and exogenous HOCl in live zebrafish and mice. More importantly, **PTA** shows remarkable effectiveness for the monitoring of HOCl‐mediated treatment response to rheumatoid arthritis.

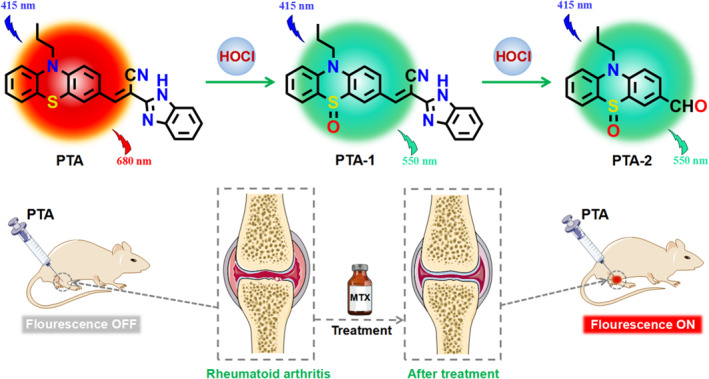

## 1 INTRODUCTION

Rheumatoid arthritis (RA) is a widespread chronic systemic inflammatory and autoimmune disease, which is mainly characterized by cartilage destruction and chronic joint synovitis. This disease seriously threatens human health for a long time and has been considered as one of the main reasons of disability and death.[^1^, ^2^, ^3^] The complicated pathogenesis of RA has not been completely understood due to the unclear etiology.[^4^, ^5^] Environmental and genetic factors may be responsible for RA.^[6]^ Recent research results have found that the excessive generation of hypochlorous acid (HOCl) is closely associated with the pathogenesis of all kinds of inflammatory diseases. Excessive HOCl generation has been deemed to be one of the signs of RA.[^6^, ^7^] HOCl is endogenously produced by myeloperoxidase‐catalyzed peroxidation of H_2_O_2_ and Cl^−^.[^8^, ^9^] Nevertheless, the disordered expression of HOCl can cause some severe diseases, including neuronal degeneration, arthritis, lung injury, rheumatoid, atherosclerosis, and even cancers.^[10]^ In addition, HOCl is usually used as an high‐efficiency disinfectant and bleach to pretreat tap water and treat industrial wastewater or industrial bleaching.^[11]^ But excessive discharge of HOCl can cause serious damage to the ecological environment and high‐dose intake can also cause some fatal diseases.[^12^, ^13^, ^14^] Accordingly, to develop high‐efficiency and accurate techniques for the detection and monitoring of HOCl in vivo has important meanings and value in the thorough understanding of the role of HOCl in RA as well as assessing the efficacy of drug therapy for HOCl‐associated diseases. Furthermore, probe‐based test papers are also needed for assessing the HOCl level in real water samples using the “naked eye.”

Compared with other detection techniques, fluorescent imaging technique has gradually developed into a promising imaging method on account of its unique advantages of high sensitivity, excellent spatiotemporal resolution, outstanding biocompatibility, and real‐time imaging, etc.[^15^, ^16^, ^17^, ^18^, ^19^, ^20^] Up to now, numerous fluorescent probes based on diverse response mechanisms have been developed, including HOCl‐induced oxidation reaction with C=C double bond,[^21^, ^22^] C=N double bond,[^10^, ^11^, ^23^] oxime,[^24^, ^25^] p‐methoxyphenol,^[26]^ p‐alkoxyaniline,[^27^, ^28^] arylmethylsulfide,[^29^, ^30^, ^31^] N,N‐dimethylthiocarbamate,[^32^, ^33^, ^34^, ^35^] the spirocyclic hydrazide group,[^36^, ^37^] arylboric acid and arylboric acid pinacol ester,[^38^, ^39^] and S atom of phenothiazine.[^40^, ^41^, ^42^] Nevertheless, most of the previously developed HOCl fluorescent probes still suffer from several problems, including shorter emission wavelengths,[^11^, ^24^, ^27^] poorer water solubility,[^10^, ^43^] and smaller Stokes shifts,[^6^, ^11^] which restrict their applications in biological systems and real water samples. Simultaneously, fluorescence chemical sensors used for the detection and monitoring of HOCl level and assessment of the treatment response in RA are also rarely reported.

**SCHEME 1:**
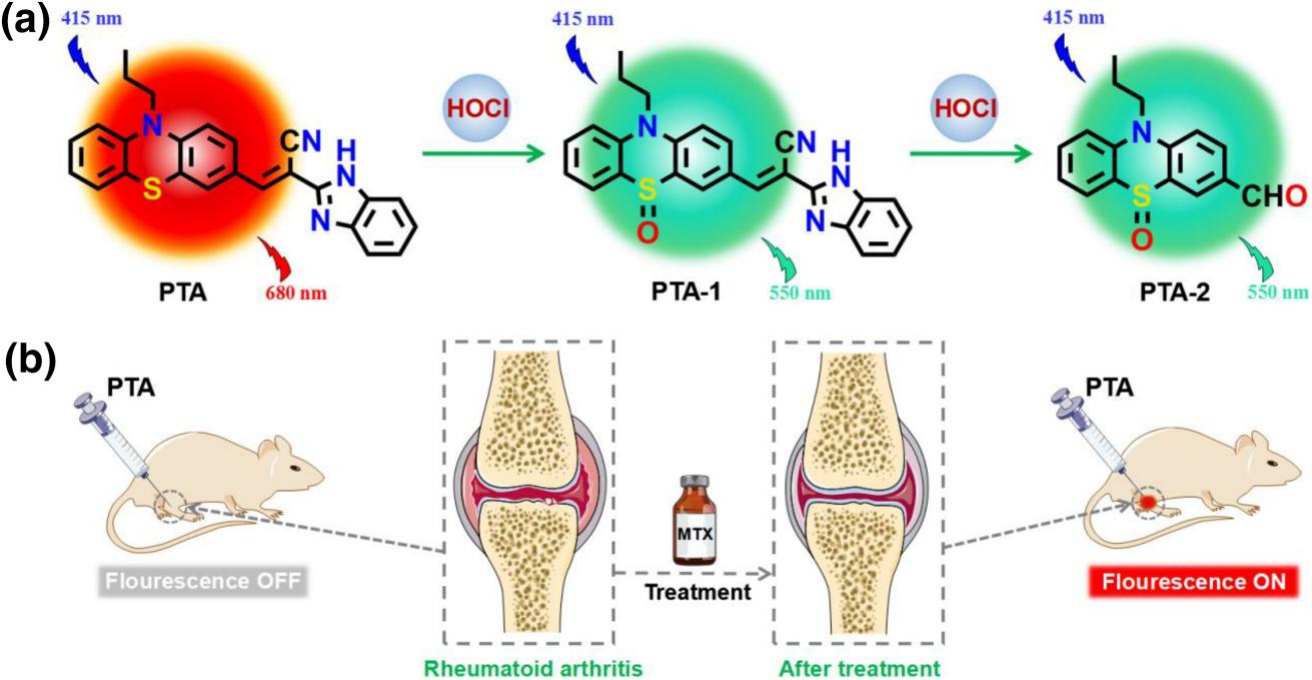
(a) Proposed response mechanisms of probe **PTA** toward HOCl. (b) The application of **PTA** in the monitoring of HOCl in an RA model. HOCl, hypochlorous acid; RA, rheumatoid arthritis.

Taking all of the aforementioned factors into account, herein, a ratiometric near‐infrared (NIR) fluorescent probe (**PTA**) was specifically designed and successfully synthesized for the detection and monitoring of HOCl in RA models and real water samples. The electron‐rich S atom and C=C bond in probe **PTA** are able to be sequentially oxidized by HOCl, leading to a remarkable hypochromatic shift of absorption spectra. At the same time, the intramolecular charge transfer (ICT) effect of **PTA** was inhibited, causing the inherent fluorescent color of **PTA** shifts from red to blue (Scheme 1a). In comparison with reported HOCl fluorescent probes (Table S4), **PTA** exhibits remarkable advantages, such as NIR emission (680 nm), ratiometric response, large Stokes shift (200 nm), high sensitivity (33.9 nM), and short response time (45 s). **PTA**‐based test paper strips were prepared and employed for the semi‐quantitative analysis of HOCl levels in real water samples using the “naked eye” assay. Particularly, **PTA** was successfully utilized for bioimaging endogenous and exogenous HOCl in live animal models, monitoring the inflammatory responses of RA in live mice, and assessing the efficacy of drug therapy for HOCl‐mediated RA (Scheme 1b).

## 2 EXPERIMENTAL SECTION

### 2.1 Synthesis of fluorescent probe PTA

**N‐Propylphenothiazine (M)** and **N‐propylphenenthiazide‐3‐formaldehyde (N)** were synthesized according to the reported synthetic route.^[43]^ N‐Propylphenenthiazide‐3‐formaldehyde (0.27 g, 1.00 mmol), 2‐(cyanomethyl)‐benzimidazole (0.16 g, 1.00 mmol), and catalyzed amount of piperidine were added in ethanol (20 mL) and refluxed for 1.5 h. After the reaction, the dark red solid obtained by thermal filtration was recrystallized in ethanol to obtain the blood red target product **PTA** with 45% yield. ^1^H NMR (400 MHz, DMSO‐*d*_*6*_) *δ* 12.94 (s, 1H), 8.18 (s, 1H), 7.83 (dd, *J* = 8.7, 1.8 Hz, 1H), 7.79 (d, *J* = 1.8 Hz, 1H), 7.60 (s, 2H), 7.21 (m, *J* = 16.2, 7.6, 5.0 Hz, 5H), 7.09 (d, *J* = 8.1 Hz, 1H), 7.01 (t, *J* = 7.4 Hz, 1H), 3.92 (t, *J* = 7.0 Hz, 2H), 1.74 (h, *J* = 7.2, 6.4 Hz, 2H), 0.97 (t, *J* = 7.3 Hz, 3H). ^13^C NMR (101 MHz, DMSO‐*d*_*6*_) *δ* 148.33, 148.07, 144.33, 143.54, 130.84, 128.48, 128.02, 127.76, 127.29, 123.92, 122.60, 117.22, 116.85, 116.37, 99.30, 49.01, 19.89, and 11.38. HR MS (positive mode, m/z), calculated for [**PTA** + H]^+^: 409.1481, found: 409.1492. Mp: 289.4–289.7°C.

### 2.2 The procedures for spectrometric analysis

Initially, the stock solution of **PTA** (in DMSO, 0.5 mM) was prepared in a 50‐mL volumetric flask. Subsequently, a 10‐μM test solution of probe **PTA** (DMSO:phosphate buffer saline [PBS] = 1:9, 20 mM, pH = 7.4) was obtained by diluting **PTA** stock solution with DMSO and PBS aqueous buffer. 20 mM stock solutions of biomolecules and anion were obtained by dissolving various biomolecules and anion salts in deionized water. Stock solutions of the ROS (including HOCl, OH, H_2_O_2_, ONOO^−^, and ^1^O_2_) were prepared in deionized water according to the reported methods.^[8]^ 2 mM NO stock solution was obtained based on methods that were previously reported.^[44]^ For spectral analysis, the test solutions were evenly mixed with the **PTA** solution (10 μM) (*V*_total_ = 3 mL) and stabilized for 5 min, then followed by fluorescence and ultraviolet spectra analyses. The excitation and emission slits of fluorescence tests were 3 and 5 nm, respectively.

### 2.3 Preparation of PTA‐based test paper strips and the application in real water samples

The filter papers (20 × 70 mm) were soaked in CH_2_Cl_2_ solution of 0.5 mM **PTA** for 15 min. **PTA**‐based test paper strips were naturally dried. Real water samples were used to dilute HOCl stock solution to obtain test solutions containing different concentrations of HOCl. The **PTA**‐based test paper strips were soaked in test solutions containing varying concentrations of HOCl for a few seconds and dried naturally. The color changes of the strips were then recorded under natural light and ultraviolet light (365 nm), respectively.

### 2.4 Visualization of exogenous and endogenous HOCl in live adult zebrafish

For the fluorescence imaging of exogenous HOCl, the adult zebrafish were incubated in **PTA‐**containing (20 μM) PBS buffer with pH = 7.4 for 5 min, and then incubated in HOCl‐containing (250 μM) PBS aqueous buffer for 1, 2, 5, 10, and 15 min, followed by fluorescence imaging.

Then, bioimaging of endogenous HOCl in zebrafish was carried out. Firstly, the adult zebrafish were pretreated with 2 μg·mL^−1^ lipopolysaccharide (LPS) for 3 h. Subsequently, the stimulated adult zebrafish were incubated in 20 μM **PTA** for 5 min.

Fluorescence imaging was performed using blank zebrafish as a control group.

### 2.5 Bioimaging of exogenous and endogenous HOCl in live mice

Nude mice were anaesthetized in isoflurane airflow in all fluorescence imaging experiments. As for bioimaging of exogenous HOCl in mice, 125 μL 20 μM **PTA** was injected subcutaneously in the left leg of the mice. Subsequently, 80 μL 250 μM HOCl was injected in the same site. Bioimaging was carried out on mice at 1, 2, 5, 10, and 15 min, respectively. Bioimaging was performed using blank mice as the control group.

As for bioimaging of endogenous HOCl in nude mice, the mice were first injected subcutaneously with 80 μL 5 μg·mL^−1^ LPS in the left leg, and then the mice were placed in a biological incubator for 5 h. Subsequently, the subcutaneous injection of 125 μL 20 μM **PTA** was administered in the same site. At the same time, the mice injected with equal amounts of **PTA** in the corresponding part of the right leg and the blank mice were the control group. Fluorescence imaging was performed on mice for 1, 5, 10, 15, and 20 min, respectively.

### 2.6 Bioimaging of HOCl in RA model

The RA model of mice was prepared according to previously reported procedures.[^3^, ^5^] Specifically, an 80 μL PBS buffer solution containing 2 μg·mL^−1^ λ‐carrageenan was injected in the joint of the left leg of mice and then the mice were placed in a biological incubator for 4 h to trigger an RA model. And, the right hind limb injected with 80 μL PBS was used as the control group. Subsequently, 125 μL 20 μM **PTA** was injected in the left leg joint with RA. In the meantime, the mice injected with equal amounts of **PTA** in the healthy right leg joint and the blank mice were the control group. Fluorescence imaging was then performed. As for the monitoring of HOCl levels in RA joints after treatment, 80 μL 2 μg·mL^−1^ anti‐arthritis drug methotrexate (MTX) was injected in the RA joints. After MTX was injected for 6 h, 125 μL 20 μM **PTA** was injected in the left and right joints and bioimaging was then performed for 10 min.

## 3 RESULTS AND DISCUSSION

### 3.1 Synthesis and sensing mechanism studies of probe PTA toward HOCl

In this research work, a ratiometric NIR **PTA** for the detection of HOCl was successfully synthesized by coupling N‐propylphenenthiazide‐3‐formaldehyde with 2‐(cyanomethyl)benzimidazole using a C=C double bond (Scheme S1). The chemical structures of intermediates (**M**, **N**) and probe **PTA** were characterized by HR‐MS and NMR (Figures S1–S9). To substantiate the proposed recognition mechanism of **PTA** toward HOCl, the mixed solution of **PTA** and HOCl was determined by HR‐MS. **PTA** revealed a strong ion peak at m/z = 409.1492 ([**PTA**+H^+^]^+^, m/z, calcd: 409.1481) (Figure S9). Nevertheless, the peak at m/z = 409.1492 disappeared completely when HOCl was added to the PTA solution. At the same time, two new molecular ion peaks assigned to **PTA‐1** ([**PTA‐1**+H^+^]^+^, m/z, calcd: 425.1431) and **PTA‐2** ([**PTA‐2**+H^+^]^+^, m/z, calcd: 286.0896) appeared at m/z = 425.1437 and m/z = 286.0898, respectively (Figure S10). The proposed sensing mechanisms, that is, sequential oxidation of S atom and C=C double bond of **PTA** induced by HOCl were preliminarily confirmed as described in Scheme 1a.

### 3.2 Theoretical calculations of probe (PTA) and oxidized products (PTA‐1 and PTA‐2)

**FIGURE 1:**
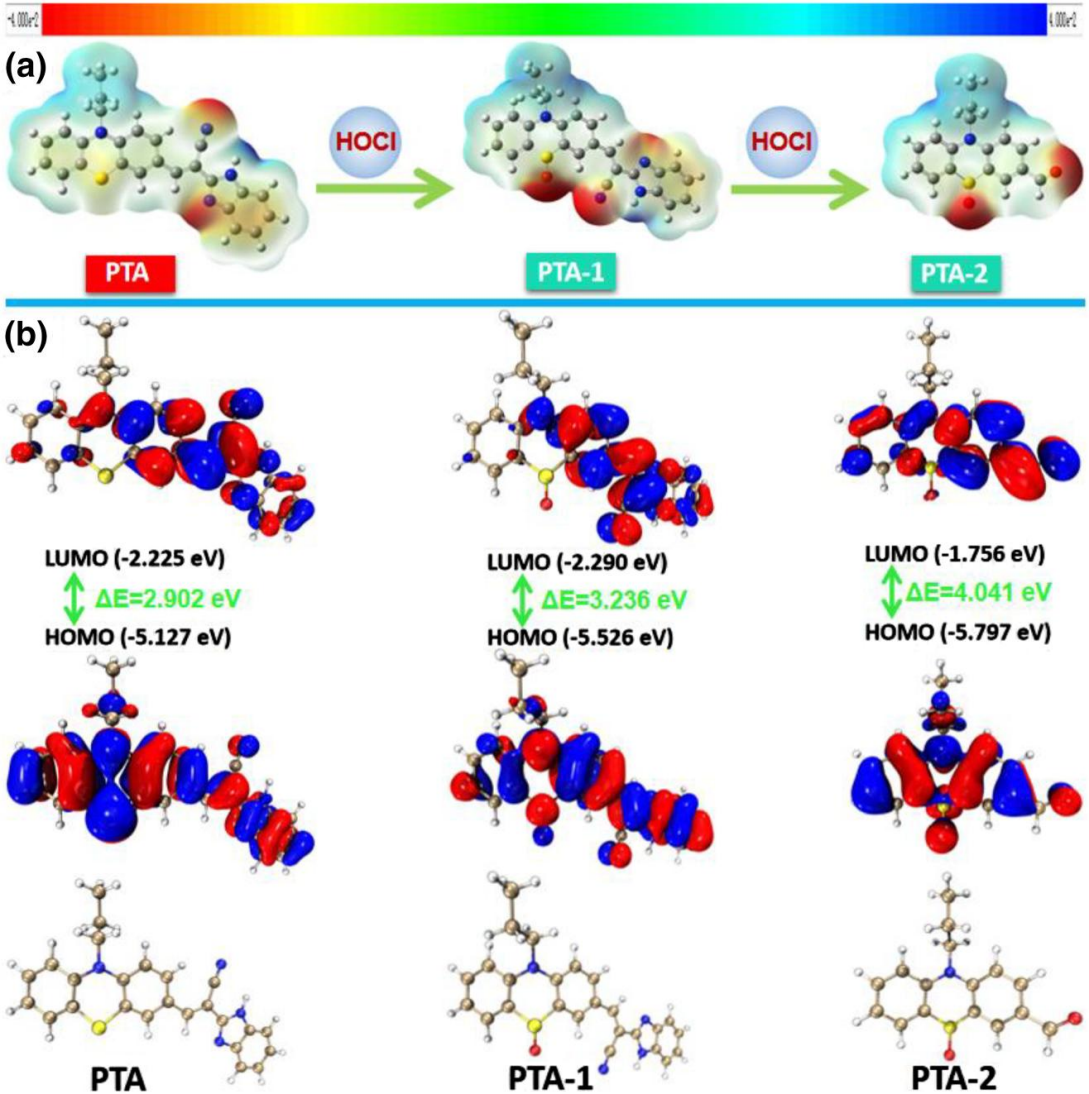
(a) Electrostatic potential surface diagram of probe (**PTA)** and oxidized products (**PTA‐1** and **PTA‐2)**. Blue and red represent positive and negative charges, respectively. (b) Optimized molecular geometry, orbital distributions of HOMO and LUMO, energy gaps of probe (**PTA)**, and oxidized products (**PTA‐1** and **PTA‐2**).

In order to clarify the response mechanisms and optical properties of probe **PTA** toward HOCl, the optimal configuration, electrostatic and HOMO and LUMO distributions of **PTA**, **PTA‐1**, and **PTA‐2** were analyzed using density functional theory (B3LYP/6‐31G (d, p) basis set) in the Gaussian 16 program. Theoretical calculations data are exhibited in Tables S1, S2, and S3. According to the maps of electrostatic potential surfaces in Figure 1a, **PTA** exhibited a significantly higher separation degree of positive and negative charges than oxidized products (**PTA‐1** and **PTA‐2**), indicating that **PTA** possesses a strong ICT effect. Subsequently, the HOMO and LUMO distributions and energy gaps of probe (**PTA)** and oxidized products (**PTA‐1** and **PTA‐2**) were compared. As provided in Figure 1b, the HOMO of **PTA** was mostly distributed on the phenothiazine group and occupies the largest fraction on S atom, while the LUMO was mainly distributed on the C=C bond and benzene ring close to the C=C bond. Hence, **PTA** has a strong ICT effect, resulting in an intense fluorescence emission at 680 nm. Nevertheless, for **PTA‐1** and **PTA‐2**, the core distances of HOMO and LUMO in **PTA‐1** and **PTA‐2** are considerably smaller than that of **PTA** due to the oxidation of electron‐rich S atom by HOCl to form sulfoxide. Therefore, the ICT effects of **PTA‐1** and **PTA‐2** are inhibited, leading to the obvious hypochromatic shift of the emission wavelengths of **PTA‐1** and **PTA‐2**. Furthermore, the HOMO‐LUMO energy gaps of **PTA‐1** (Δ*E* = 3.236 eV) and **PTA‐2** (Δ*E* = 4.041 eV) are smaller than that of **PTA** (Δ*E* = 2.902 eV), which is also consistent with the experimental phenomenon that the emission wavelengths of **PTA‐1** and **PTA‐2** are smaller than that of **PTA**.

### 3.3 UV‐vis spectral response of PTA to HOCl

**FIGURE 2:**
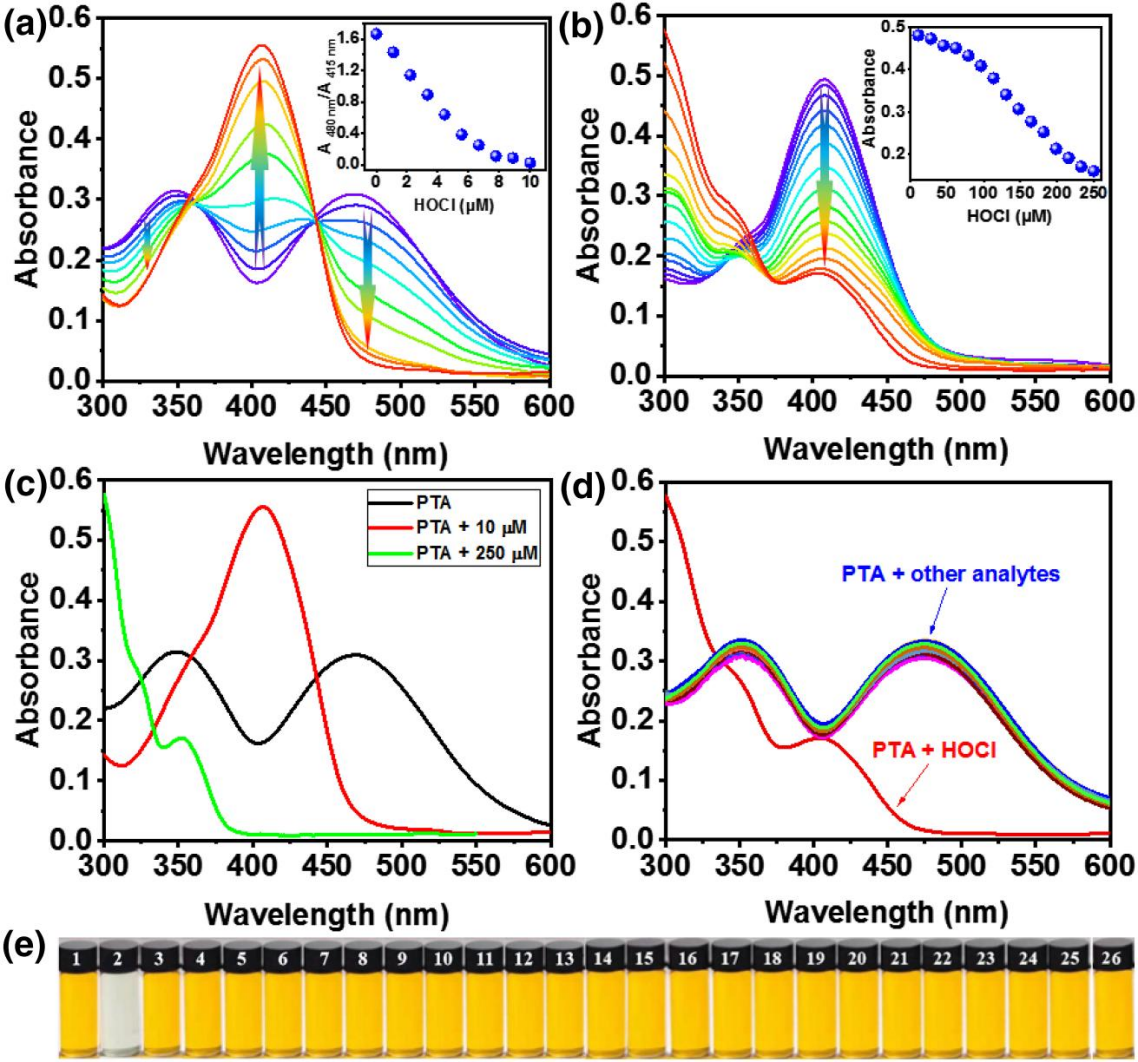
(a) UV‐vis spectra of 10 μM **PTA** in coexistence with HOCl (0–10 μM) in PBS buffer solution with pH = 7.4. Inset: The ratios of the absorbances (A_480 nm_/A_415 nm_) as a function of HOCl concentrations (0–10 μM). (b) Absorption spectra of 10 μM **PTA** in coexistence with HOCl (11–250 μM). Inset: Absorbance at 410 nm as a function of HOCl concentrations (11–250 μM). (c) UV‐vis spectra of 10 μM **PTA** in coexistence with HOCl (0, 10, and 250 μM). (d) Ultraviolet spectra responses and (e) color responses of 10 μM **PTA** to multifarious analytes (250 μM), including 1. Only **PTA**, 2. HOCl, 3. HSO_3_^−^, 4. HCO_3_^−^, 5. Cl^−^, 6. SO_4_^2−^, 7. OH^−^, 8. Br^−^, 9. S^2−^, 10. NO_2_^−^, 11. HSO_4_^−^, 12. PO_4_^3−^, 13. P_2_O_7_^4−^, 14. AcO^−^, 15. F^−^, 16. NO_3_^−^, 17. H_2_PO_4_^−^, 18. SO_3_^2−^, 19. ·OH, 20. H_2_O_2_, 21. ONOO^−^, 22. ^1^O_2_, 23. Cys, 24. GSH, 25. Hcy, and NO. HOCl, hypochlorous acid.

Initially, UV‐vis spectral titration of **PTA** to HOCl was investigated in PBS buffer solution with pH = 7.4. **PTA** displayed two absorption bands at 355 and 480 nm, respectively (Figure 2a). The two absorption peaks declined gradually on the increase of HOCl (0−10 μM) concentration, while the absorption band 415 nm continued to strengthen. Absorbance ratios (A_480 nm_/A_415 nm_) decreased significantly and the maximum absorption wavelength was blue shifted by 65 nm (Figure 2a, inset). The experimental phenomenons can be attributed to the oxidation of the sulfur atom of phenothiazine to the sulfoxide structure (**PTA‐1**) within 1.0 equiv. HOCl. Whereas, the ultraviolet absorption band centered at 415 nm decreased homogeneously when HOCl (11−250 μM) concentration increased further (Figure 2b). And the absorption peak remained unchanged when the concentration of HOCl peaked at 250 μM (Figure 2b, inset). In this process, the introduction of HOCl led to the oxidation of the C=C of **PTA‐1** to form **PTA‐2** (Scheme 1a). Hence, the absorption spectrum of **PTA** was significantly affected by HOCl concentrations (0, 10, and 250 μM) (Figure 2c). The results of comprehensive absorption spectra analysis indicated that the reaction of probe **PTA** toward HOCl possesses concentration selective properties. Subsequently, the specific recognition of **PTA** for HOCl was further explored. As displayed in Figure 2d, UV‐vis spectra of **PTA** declined dramatically in the presence of HOCl (250 μM), while no significant change was observed when **PTA** coexisted with other analytes (250 μM), including HSO_3_^−^, HCO_3_^−^, Cl^−^, SO_4_^2−^, OH^−^, Br^−^, S^2−^, NO_2_^−^, HSO_4_^−^, PO_4_^3−^, P_2_O_7_^4−^, AcO^−^, F^−^, NO_3_^−^, H_2_PO_4_^−^, SO_3_^2−^, OH, H_2_O_2_, ONOO^−^, ^1^O_2_, Cys, GSH, Hcy, and NO. Additionally, the specific recognition of **PTA** for HOCl was also substantiated using “naked eye” colorimetry. The solution color of **PTA** changes from yellow to colorless only in the presence of HOCl, demonstrating that **PTA** had high selectivity toward HOCl (Figure 2e).

### 3.4 Fluorescence spectral response of PTA to HOCl

**FIGURE 3:**
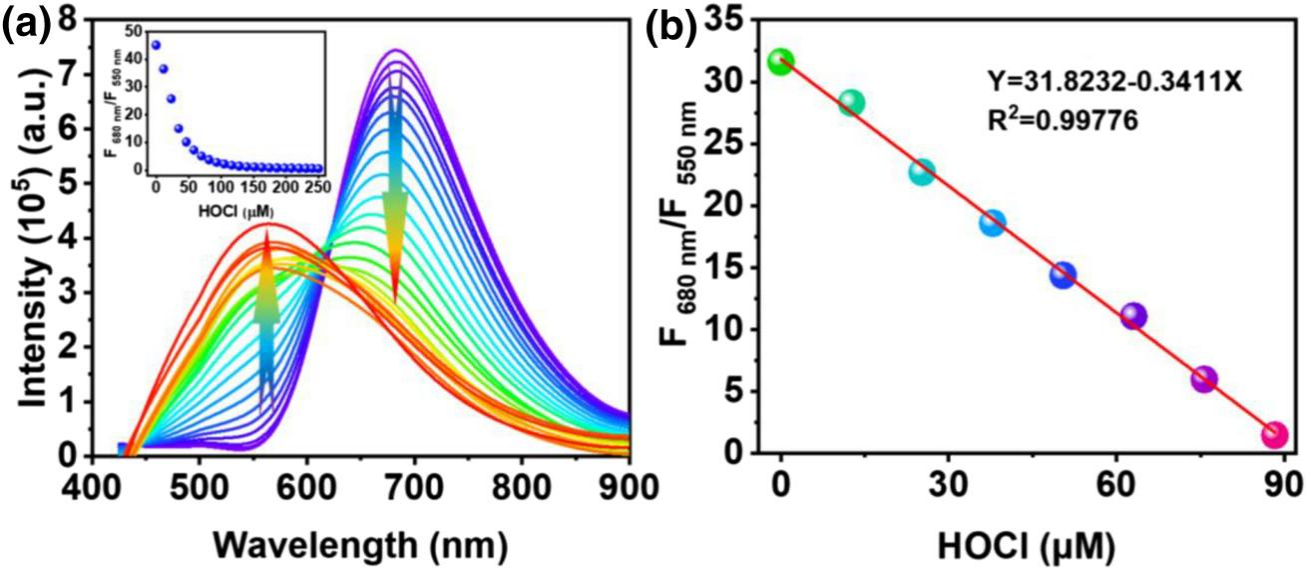
(a) Emission spectra of 10 μM **PTA** in coexistence with HOCl (0–250 μM) in PBS buffer solution with pH = 7.4. Inset: Emission intensity ratios at 680 and 550 nm as a function of HOCl concentrations (0–250 μM). (b) The linearity curve between the emission intensity ratios of 5 μM **PTA** at 680 and 550 nm and HOCl concentrations (0–88.33 μM). *λ*_*ex*_ = 415 nm. HOCl, hypochlorous acid.

The responses of **PTA** to HOCl were further investigated by emission spectra titration. Firstly, kinetic stability of **PTA** was assessed by measuring the changes of fluorescence intensities with time. Emission intensities of **PTA** at 680 and 550 nm remained unchanged within 45 h in PBS aqueous buffer with pH = 7.4 (Figure S11), demonstrating that **PTA** had high stability. Subsequently, the emission spectral response of **PTA** to HOCl was investigated. As demonstrated in Figure 3a, **PTA** displayed an intense emission at 680 nm. Emission intensities of **PTA** at 680 nm gradually decreased with increasing HOCl concentration (0–250 μM), accompanied by the gradual increase of emission intensities at 550 nm. Emission intensity ratios (F_680 nm_/F_550 nm_) decreased to the minimum when HOCl concentration increased to 250 μM (Figure 3a, inset). In addition, the ratios of the fluorescence intensities (F_680 nm_/F_550 nm_) exhibited a perfect linearity (*R* ^2^ = 0.99776) to the HOCl (0–88.33 μM) concentrations (Figure 3b). The detection limit of **PTA** to HOCl is 33.9 nM according to the formula defined by IUPAC.^[45]^

**FIGURE 4:**
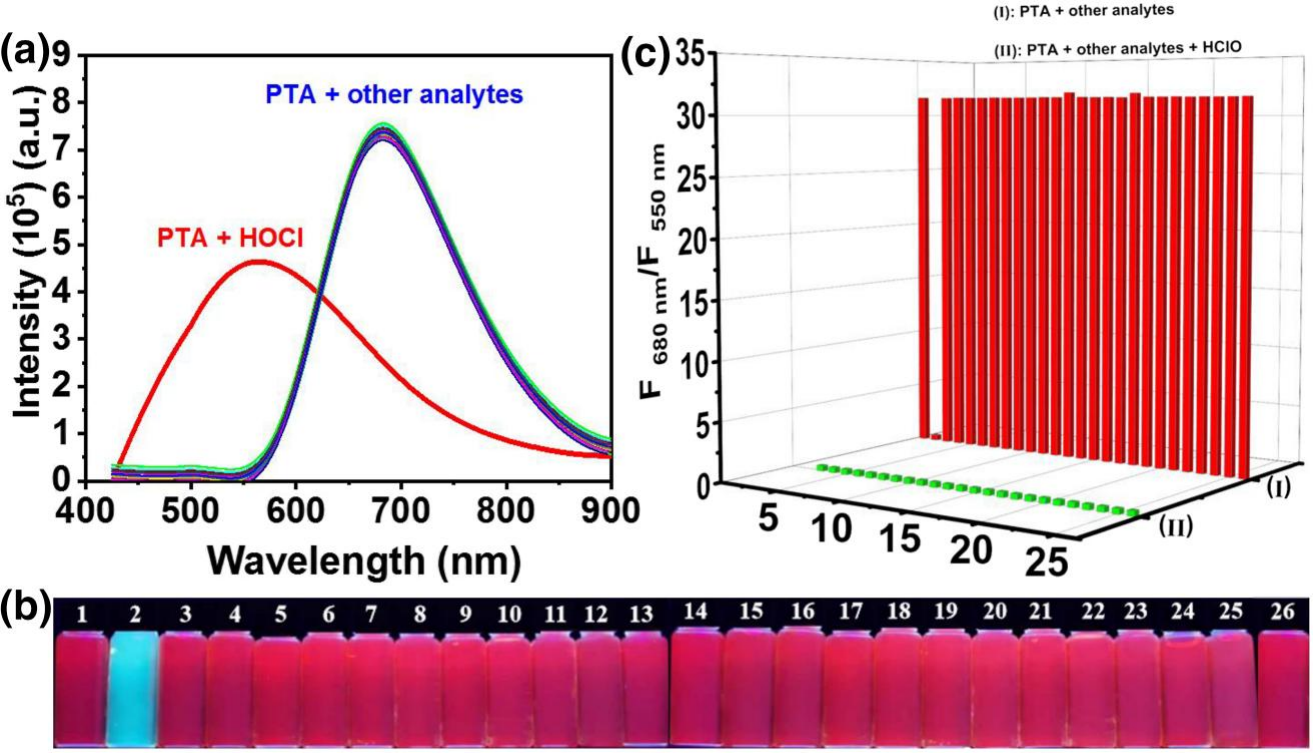
(a) Emission spectral responses and (b) fluorescent color recognition under 365 nm UV light of **PTA** (10 μM) toward various analytes (250 μM) including 1. Only **PTA**, 2. HOCl, 3. HSO_3_^−^, 4. HCO_3_^−^, 5. Cl^−^, 6. SO_4_^2−^, 7. OH^−^, 8. Br^−^, 9. S^2−^, 10. NO_2_^−^, 11. HSO_4_^−^, 12. PO_4_^3−^, 13. P_2_O_7_^4−^, 14. AcO^−^, 15. F^−^, 16. NO_3_^−^, 17. H_2_PO_4_^−^, 18. SO_3_^2−^, 19. OH, 20. H_2_O_2_, 21. ONOO^−^, 22. ^1^O_2_, 23. Cys, 24. GSH, 25. Hcy, and NO. (c) Emission responses of **PTA** (10 μM) to HOCl (250 μM) when coexisting with aforementioned competitive analytes (250 μM). *λ*_*ex*_ = 415 nm, *λ*_*em*_ = 680 nm, and 550 nm.

The specific recognition of **PTA** to HOCl was further explored using fluorescence spectra analyses. As displayed in Figure 4a, the emission spectra remained unchanged when **PTA** coexisted with other analytes (250 μM) such as HSO_3_^−^, HCO_3_^−^, Cl^−^, SO_4_^2−^, OH^−^, Br^−^, S^2−^, NO_2_^−^, HSO_4_^−^, PO_4_^3−^, P_2_O_7_^4−^, AcO^−^, F^−^, NO_3_^−^, H_2_PO_4_^−^, SO_3_^2−^, ·OH, H_2_O_2_, ONOO^−^, ^1^O_2_, Cys, GSH, Hcy, and NO. However, the emission spectra changed significantly after adding HOCl exclusively. In the meantime, the specific recognition of **PTA** toward HOCl was also demonstrated using “naked eye” fluorescent colorimetry. The red fluorescence color of **PTA** turns to blue only in coexistence with HOCl (Figure 4b). Furthermore, the specific recognition of **PTA** to HOCl was further investigated in coexistence with other interference analytes (250 μM). The emission response of **PTA** toward HOCl in coexistence with other competitive analytes is similar to that of **PTA** toward HOCl only (Figure 4c). The aforementioned experiments demonstrated that **PTA** possesses a high specificity to HOCl and could be employed for the recognition of HOCl in complex systems.

### 3.5 pH/time‐dependent emission responses of PTA to HOCl

Furthermore, the effect of pH on the recognition of **PTA** toward HOCl were explored. As presented in Figure S12, the ratios of the fluorescence intensities (F_680 nm_/F_550 nm_) of **PTA** were stable in the range of pH 5.0–10.0. After HOCl was added, the ratios of **PTA** decreased observably within the range of pH 4.5–10.0. Thus, probe **PTA** can be utilized for the detection of HOCl in a broad range of pH and is available under physiological conditions. To explore the response time of **PTA** toward HOCl, time‐dependent emission response of **PTA** to HOCl at 680 nm was determined in PBS aqueous buffer with pH = 7.4. **PTA** exhibited strong and stable fluorescence at 680 nm in PBS aqueous buffer (Figure S13). The emission intensities of **PTA** decreased promptly when HOCl (250 μM) was employed and reached the lowest within 45 s. The experimental result demonstrates that the dynamic response of **PTA** to HOCl is quite fast.

### 3.6 PTA‐based test paper strips for the determination of HOCl in water samples

**FIGURE 5:**
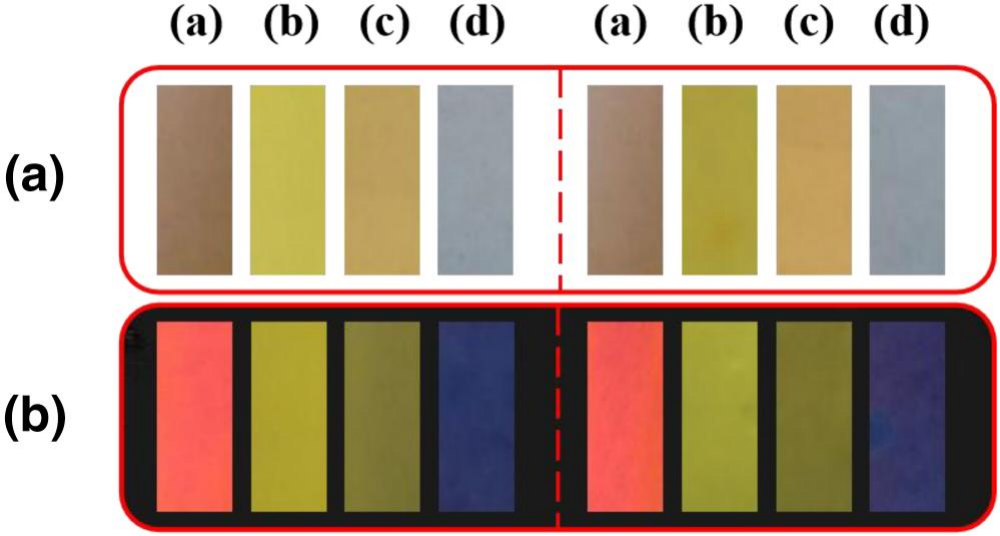
Photographs of the **PTA**‐based test paper strips were immersed in different water samples containing HOCl of different concentrations, including (a) 0 mM, (b) 0.10 mM, (c) 0.50 mM, and (d) 1.00 mM. The four groups on the left are drinking water samples and the four groups on the right are tap water samples. (a) Photographs under natural light conditions and (b) Photographs under 365 nm UV light. HOCl, hypochlorous acid.

Then, **PTA** was prepared as test paper strips and used for the rapid determination of HOCl level in real water samples. As displayed in Figure 5, the **PTA**‐based test paper strip showed brown color under natural light and intense red fluorescence under UV light (365 nm). In order to detect HOCl in real water samples, **PTA**‐based test paper strips were soaked in drinking water or tap water samples for 5.0 min. Subsequently, changes of the fluorescence color and color of the test strips were imaged. With the continuous increase in the concentration of HOCl (0–1.0 mM), the fluorescent colors of **PTA**‐based test strips changed from strong red to yellow and finally to no fluorescence. Simultaneously, the color of the **PTA**‐based test paper strips changed from brown to colorless. The study results demonstrated that the **PTA**‐based test paper strip is feasible for convenient and rapid determination of HOCl in real water samples.

### 3.7 Imaging of HOCl in vivo

Before performing imaging of HOCl in vivo, the cytotoxicity of **PTA** was preferentially tested using a standard MTT assay.^[46]^ After incubating the A549 cells with **PTA** (10 and 20 μM) for 24 h, the viability of A549 cells was maintained at a high level (94.67% and 90.82%), respectively (Figure S14). The results manifested that probe **PTA** has low cytotoxicity. In the first place, the in vivo imaging ability of **PTA** toward exogenous HOCl was investigated utilizing adult zebrafish as a vertebrate model. As manifested in Figure S15, no significant fluorescence signal was found in the untreated zebrafish. Nevertheless, after incubating with **PTA** (20 μM) for 5 min, the abdomen and gills of adult zebrafish exhibited a strong fluorescence signal, indicating the effective uptake of probe **PTA** by adult zebrafish. Subsequently, the **PTA**‐pretreated zebrafish were further incubated with HOCl (250 μM), and it can be clearly seen that the signal intensities in the injection areas weakened gradually with the increase in incubation time and disappeared entirely until the incubation time reached 15 min. Then, the capability of **PTA** for fluorescence imaging of endogenous HOCl in vivo was explored in zebrafish. **PTA**‐incubated zebrafish shows strong fluorescence signals in the abdomen and gills, while no obvious fluorescence signals were found in both zebrafish stimulated with 2 μg·mL^−1^ LPS and untreated adult zebrafish (Figure S16). However, after LPS‐stimulated zebrafish were further treated with 20 μM **PTA** for 5 min, a silent emission signal was observed. The above experiments presented that **PTA** can be employed for fluorescence bioimaging of exogenous and endogenous HOCl in zebrafish.

**FIGURE 6:**
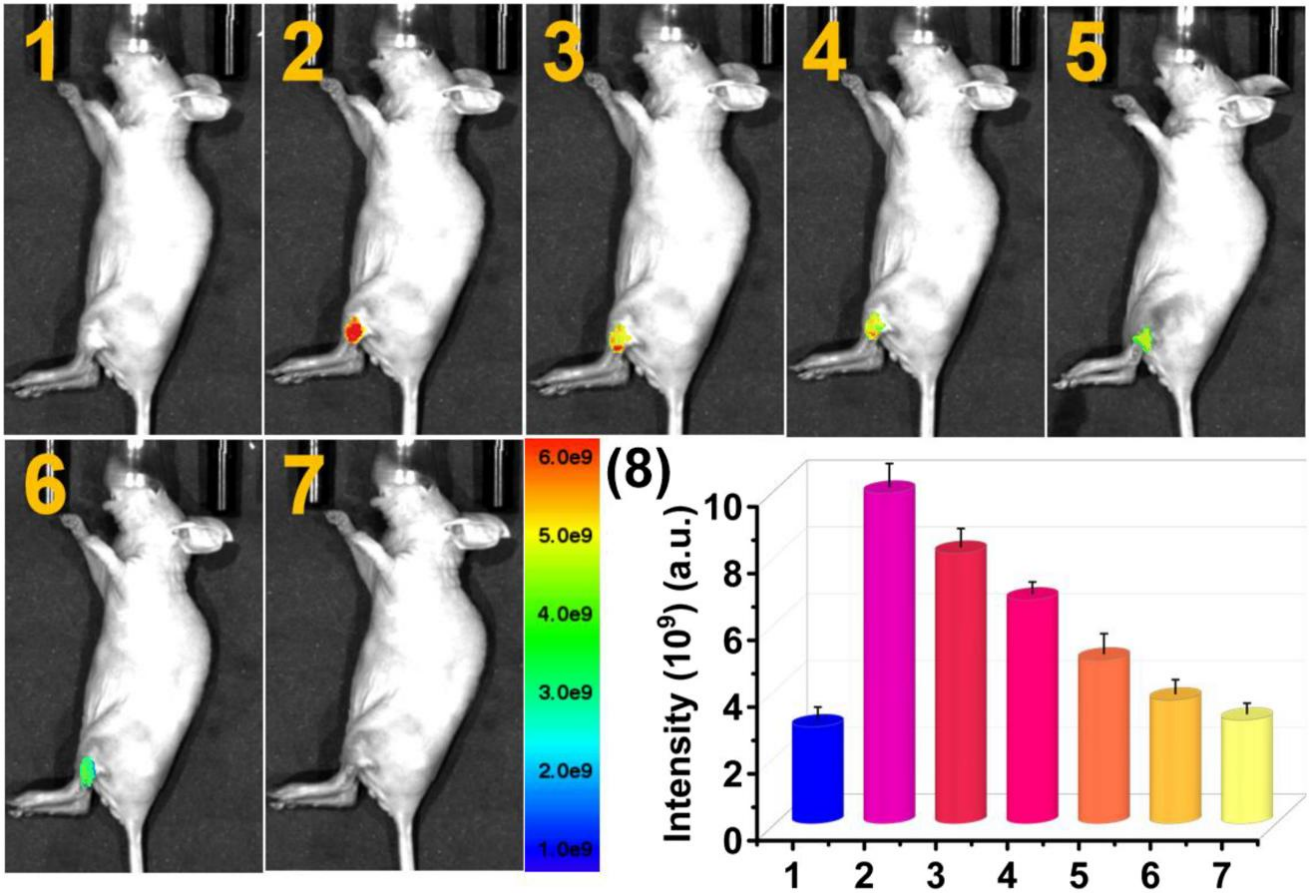
Detection of exogenous HOCl in live mice. (1) Blank mice and (2) probe **PTA** (20 μM, 125 μL) was injected into the leg of mice. Subsequently, 80 μL 250 μM HOCl was injected into the same site and bioimaging was performed at (3) 1.0 min, (4) 2.0 min, (5) 5.0 min, (6) 10.0 min, and (7) 15.0 min, respectively. (8) The average intensities at the injection site of the mouse leg in (1–7). Imaging studies were performed on mice using 500 nm excitation filter and 660 nm emission filter. HOCl, hypochlorous acid.

The feasibility of **PTA** to in vivo image exogenous HOCl was also evaluated in live mice. As presented in Figure 6, after subcutaneous injection of 125 μL 20 μM **PTA** in the left hind leg of nude mice (6–8 weeks of age), a strong fluorescence signal was displayed at the injection area. However, after continuously injecting 80 μL 250 μM HOCl at the same site, the fluorescence intensities at the injection area decreased gradually over time and disappeared completely when the time reached 15 min, demonstrating that **PTA** could be employed to image exogenous HOCl in live mice.

**FIGURE 7:**
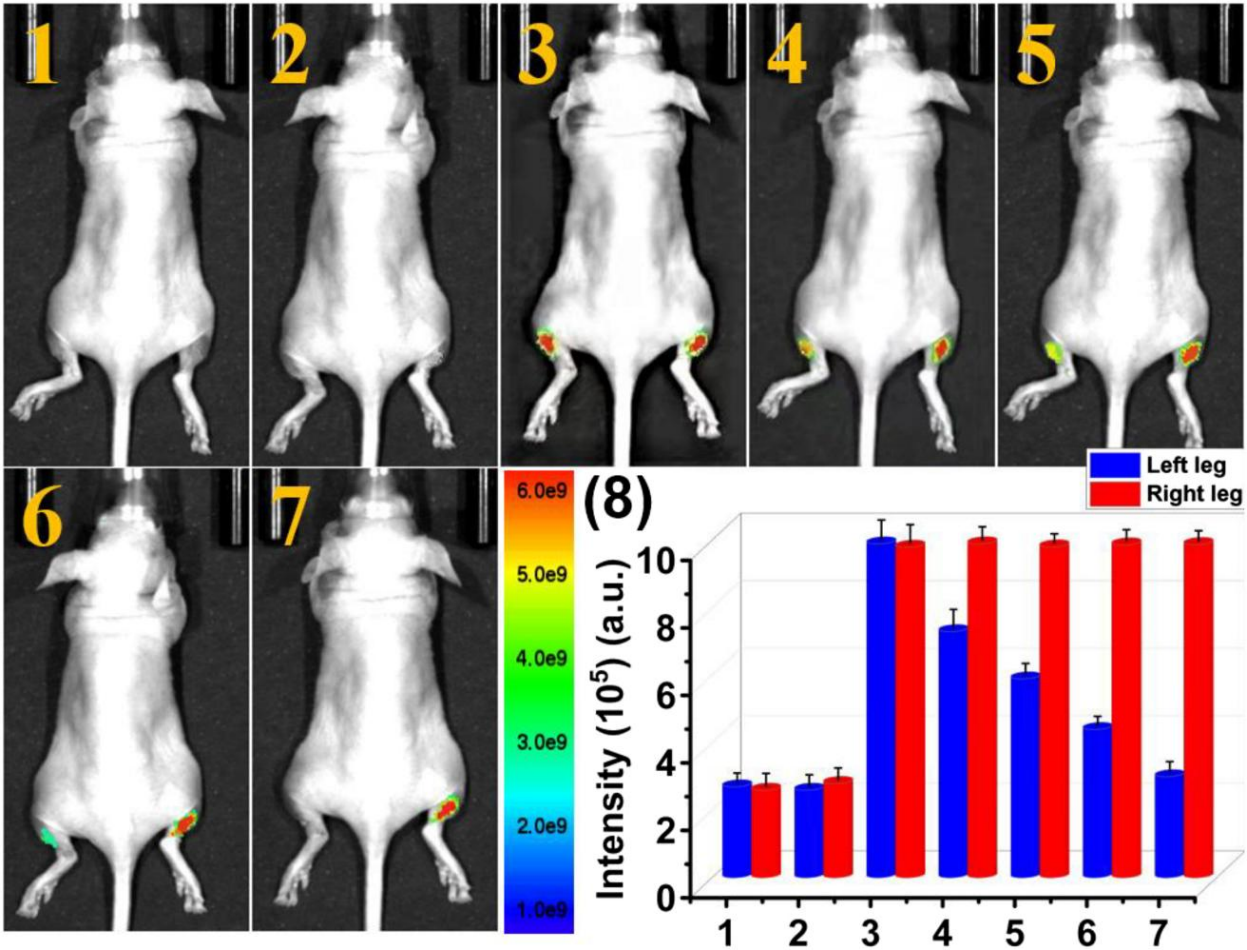
Visualization of endogenous HOCl in live mice by fluorescence imaging. (1) Blank mice and (2) the left leg of mice was first injected with 80 μL 5 μg ml^−1^ LPS and then the mice were placed in a biological incubator for 5 h. Subsequently, 125 μL 20 μM **PTA** was injected into the same site and imaging was performed at (3) 1 min, (4) 5 min, (5) 10 min, (6) 15 min, and (7) 20 min, respectively. Equal amount of **PTA** was injected in the right leg as the control group. (8) The average intensities at the injection site of the mouse leg in (1–7). Imaging studies were performed on mice using 500 nm excitation filter and 660 nm emission filter. HOCl, hypochlorous acid.

Subsequently, we investigated the fluorescence imaging ability of **PTA** toward endogenous HOCl in nude mice. As displayed in Figure 7, after the injection of 80 μL 5 μg·mL^−1^ LPS and PBS on the left and right legs of mice for 5 h, respectively, no remarkable fluorescence was revealed at the injection site. Then, 125 μL 20 μM **PTA** was injected at the same injection sites of the left and right legs, respectively. Initially, intense fluorescence signals were observed at the injection sites of both hind legs. As time goes on, the signal intensity basically unchanged in the LPS‐untreated right leg. However, the emission intensities of the left leg gradually decreased with the passage of time and disappeared when the time reached 20 min.

**FIGURE 8:**
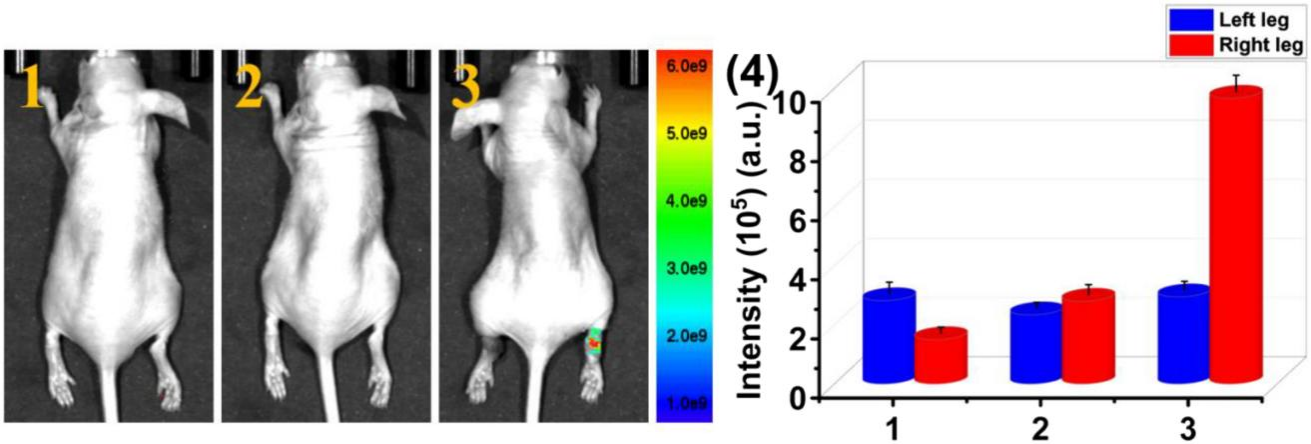
Visualization of HOCl in the RA model. (1) Blank mice and (2) 80 μL 2 μg·mL^−1^ λ‐carrageenan was injected in the left hind leg joint of mice and then the mice were placed in a biological incubator for 4 h. (3) 125 μL 20 μM **PTA** was injected in the joints of left and right hind legs. (4) The average intensities at the injection site of the mouse leg in (1–3). Imaging studies were performed on mice using 500 nm excitation filter and 660 nm emission filter. HOCl, hypochlorous acid; RA, rheumatoid arthritis.

We further investigated the feasibility of **PTA** for visualization of endogenous HOCl in the RA model by fluorescence imaging assay. 80 μL 2 μg·mL^−1^ λ‐Carrageenan was firstly injected in the left limb of the mice and incubated for 4 h to generate the RA model. The right limb was injected with 80 μL PBS (control group). Then, 125 μL 20 μM **PTA** were injected in the same sites of the joints of both left and right hind legs, respectively. A negligible signal was observed in the RA‐generated left joint (Figure 8), which indicated that **PTA** reacts with the endogenous HOCl to quench the fluorescence signal collected at 660 nm. However, strong fluorescence was still maintained in the right joint arising from no endogenous HOCl generation in the healthy right joint. The results sufficiently supported the fact that **PTA** is capable of imaging endogenous HOCl in the RA area.

**FIGURE 9:**
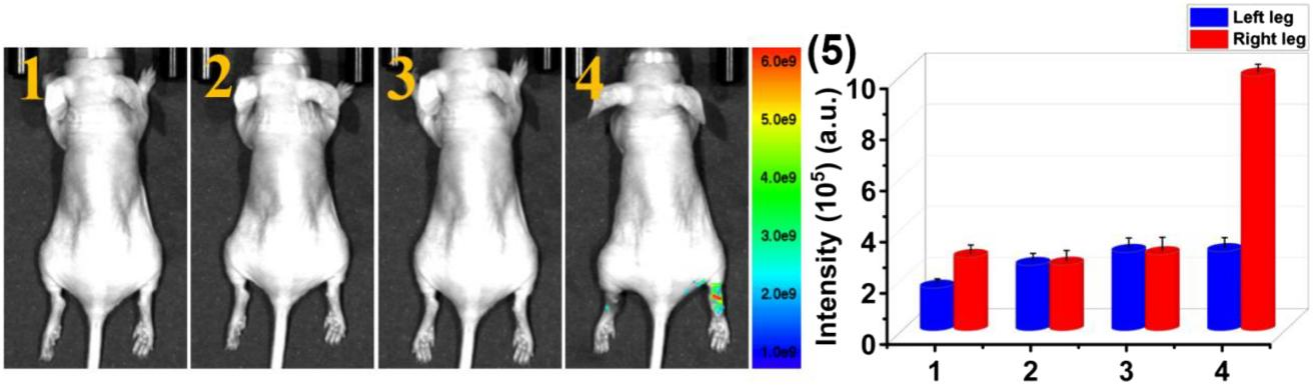
Monitoring of HOCl in RA model treatment response. (1) Blank mice and (2) 80 μL 2 μg·mL^−1^ λ‐carrageenan was injected in the joints of both left and right legs of mice, and the mice were then placed in a biological incubator for 4 h, (3) 80 μL 2 μg ml^−1^ MTX was injected in the right joints of mice for another 6 h. (4) 125 μL 20 μM **PTA** was injected in the joints of both left and right legs of mice. (5) The average intensities at the injection site of the mouse leg in (1–4). Imaging studies were performed on mice using 500 nm excitation filter and 660 nm emission filter. HOCl, hypochlorous acid; RA, rheumatoid arthritis.

Finally, the ability of **PTA** to assess HOCl‐mediated RA therapy in live mice has been further evaluated. As presented in Figure 9, after RA‐generated right leg joint was treated with MTX, a common treatment drug for RA, 125 μL 20 μM **PTA** was then injected into the joints of both left and right legs of mice. A significant fluorescence signal appeared at the injection site of the right leg joint that was treated with MTX, which demonstrated that the generation of HOCl is inhibited during the treatment of RA. In contrast, the left leg joint that has not received MTX treatment, the endogenous HOCl in RA area still quenched the fluorescence emission collected at 660 nm of **PTA**. The results confirmed that **PTA** could be used for monitoring the treatment of RA by the detection of changes in HOCl levels.

## 4 CONCLUSIONS

In summary, an NIR fluorescent probe (**PTA**) was successfully developed for the detection and monitoring of HOCl with a ratiometric response model. In the presence of 1.0 equiv. of HOCl, the electron‐rich S atom of probe **PTA** was preferentially oxidized to sulfoxide structure (**PTA‐1**), leading to significant hypochromatic shift of absorption spectra. Nevertheless, in the presence of higher concentrations of HOCl, the C=C double bond of **PTA‐1** was sequentially oxidized to form carbaldehyde derivative (**PTA‐2**), leading to the absorption peak of **PTA** centered at 415 nm declined dramatically. Furthermore, the ICT process of **PTA** was inhibited after continuous oxidation of **PTA** by HOCl, which led to the hypochromatic shift of the inherent red fluorescence of **PTA**. The **PTA**‐based test paper strips were prepared and successfully applied to determinate HOCl in actual water samples using the “naked eye” colorimetric method. It is worth mentioning that **PTA** exhibits remarkable advantages, including NIR emission, large Stokes shift, low cytotoxicity, high sensitivity, and short response time. **PTA** has been successfully utilized for in vivo imaging of endogenous and exogenous HOCl in living zebrafish and mice. More importantly, HOCl‐mediated RA treatment response using **PTA** as a fluorescence imaging probe was successfully realized. As a result, **PTA** provides a new approach to further understand the role of HOCl in RA and to evaluate the drug treatment efficiency of RA, which provides a flexible strategy for broad applications in medical diagnosis.

## CONFLICT OF INTEREST STATEMENT

The authors declare no conflict of interest.

## ETHICS STATEMENT

All animal procedures were performed in accordance with the Guidelines for Care and Use of Laboratory Animal and experiments were approved by the Animal Ethics Committee of University of Science and Technology Liaoning.

[Correction added on 01 April 2023, after first online publication: Ethics Statement has been added.]

## Supporting information

Supporting Information S1

## Data Availability

The data that support the findings of this study are available from the corresponding author upon reasonable request.
