# Supplementary material for: A ratiometric near‐infrared fluorescent probe for the detection and monitoring of hypochlorous acid in rheumatoid arthritis model and real water samples
Source: Smart Mol. 2023 Mar 21;1(2):e20220007. doi: 10.1002/smo.20220007 (PMC12118246; doi:10.1002/smo.20220007)
Supplement: Supplementary file 1 — Supporting Information S1 [file SMO2-1-e20220007-s001.docx]

Supplementary Information

**A Ratiometric** **Near-infrared Fluorescent Probe for the Detection and Monitoring of Hypochlorous Acid in Rheumatoid Arthritis Model and Real Water Samples**

Zhuye Shang‡^a^, Xinyi Yang‡^a^, Qingtao Meng^a,b^*, Shengye Tian^a^, Zhiqiang Zhang^a,^*

^a^ School of Chemical Engineering, University of Science and Technology Liaoning, Anshan, Liaoning Province, 114051, P. R. China.

E-mail: [qtmeng@ustl.edu.cn](mailto:qtmeng@ustl.edu.cn) (Q. T. Meng), [zzq@ustl.edu.cn (Z. Q. Zhang),](mailto:zzq@ustl.edu.cn%20(Z.%20Z.%20Zhang);) Tel.: +86-412-5929637

^b^ Key Laboratory for Functional Material, Educational Department of Liaoning Province, University of Science and Technology Liaoning, Anshan, Liaoning Province 114051, PR China.

‡ These authors contributed equally to this work and they should be regarded as co-first authors.

**Reagents and materials**

Phenothiazine, 1-bromopropane, sodium hydride and 2-(cyanomethyl)benzimidazole were received from Aladdin reagent Co. (Shanghai, China). Piperidine, phosphorus oxychloride (POCl_3_), hydrogen peroxide (H_2_O_2_), anions (sodium salts) and sodium hypochlorite (NaOCl) were purchased from Sinopharm Chemical Reagent Co., Ltd. (China). All of the experimental live zebrafish and nude mice were obtained from Liaoning Changsheng Biotechnology Co., Ltd., and the experiments were performed in compliance with the relevant laws and guidelines. Unless otherwise stated, solvents and reagents were of analytical grade from commercial suppliers and were used without further purification.

**Apparatus**

^1^H NMR and ^13^C NMR spectra were recorded with an AVANCE 400 MH_Z_ and 600 MH_Z_ spectrometer (Bruker) with chemical shifts reported as ppm (in CDCl_3_ and DMSO-*d*_6_, TMS as an internal standard). Atmospheric pressure ionization (API) mass spectra were recorded on an Agilent 6530 QTOF spectrometer. Absorption spectra were tested with a Perkin Elmer Lambda 900 UV/VIS/NIR spectrophotometer. Fluorescence spectra were recorded with a Spectrofluorometer FS5 luminescence spectrometer. All pH measurements were made with an OHAUS Starter 3100/f meter. Imaging of HOCl in adult zebrafish and mice were performed on a Spectral Ami Imaging Systems (Spectral Instruments Imaging, LLC, Tucson, AZ) with an excitation filter 500 nm and an emission filter 660 nm and all data for fluorescence imaging were acquired and processed using Amiview Living Image 2.0 software (PerkinElmer, USA).

MTT assay of cytotoxicity of PTA in A549 cells

The cytotoxicity of **PTA** to live A549 cells was investigated by MTT assays.^1^ The following formula was used to calculate the viability of cell growth: Viability (%)=(mean of absorbance value of treatment group-blank)/(mean absorbance value of control-blank)×100.

**Scheme S1.** Synthetic procedure of probe **PTA**.


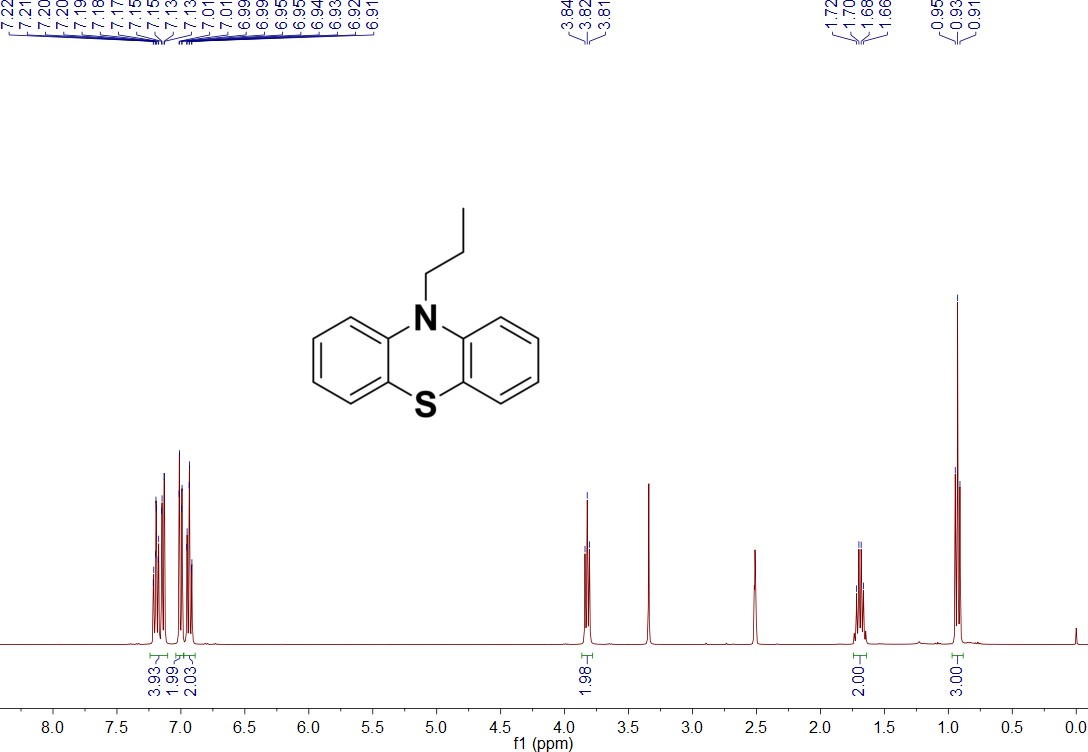


**Fig. S1.** ^1^H NMR of N-propylphenothiazine (DMSO-*d*_6_, 400 MHz).


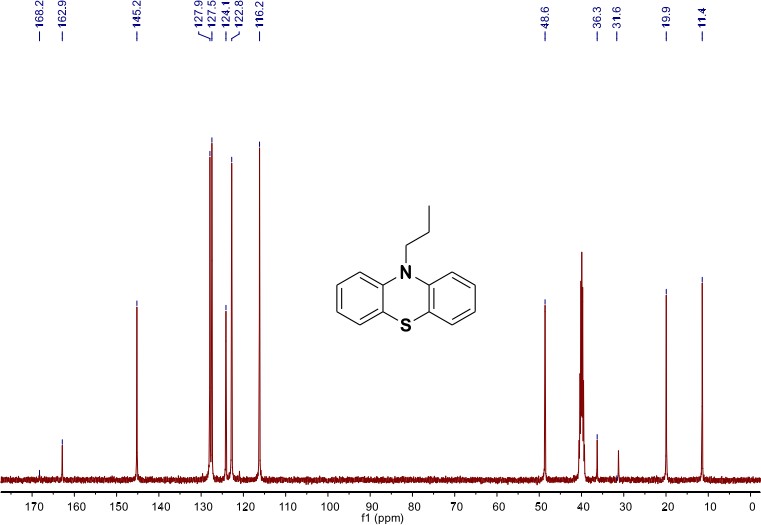


**Fig. S2.** ^13^C NMR of N-propylphenothiazine (DMSO-*d*_6_, 101 MHz).


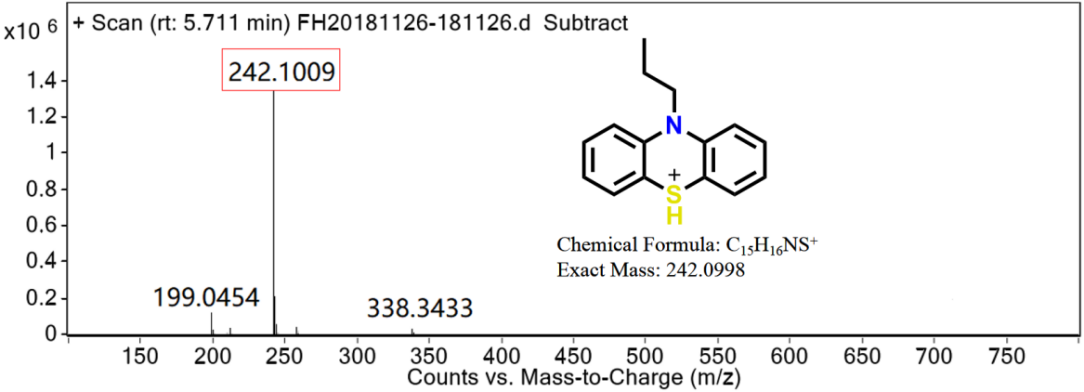


**Fig. S3.** HR-MS of N-propylphenothiazine.


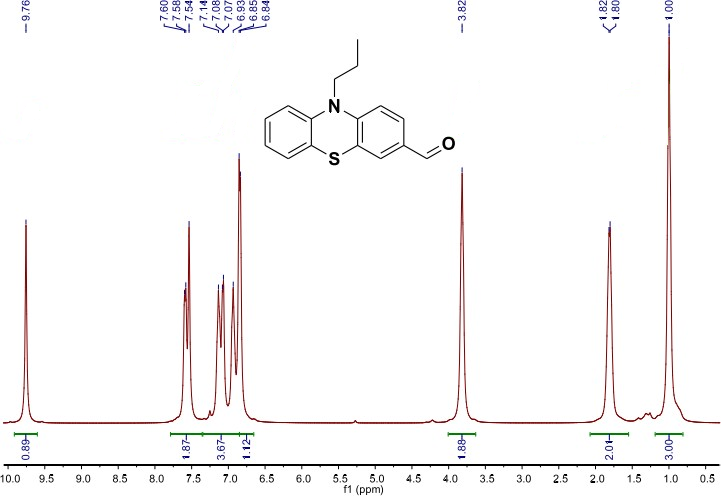


**Fig. S4.** ^1^H NMR of N-propylphenothiazine-3-formaldehyde (CDCl_3_, 600 MHz).


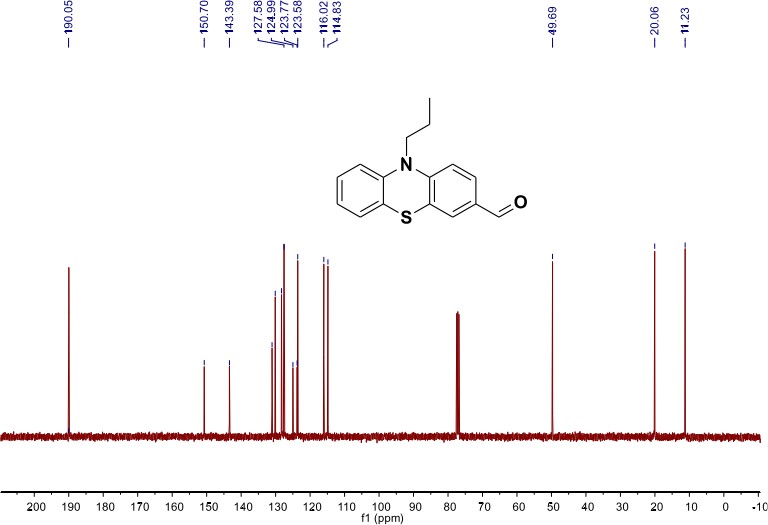


**Fig. S5.** ^13^C NMR of N-propylphenothiazine-3-formaldehyde (CDCl_3_, 151 MHz).


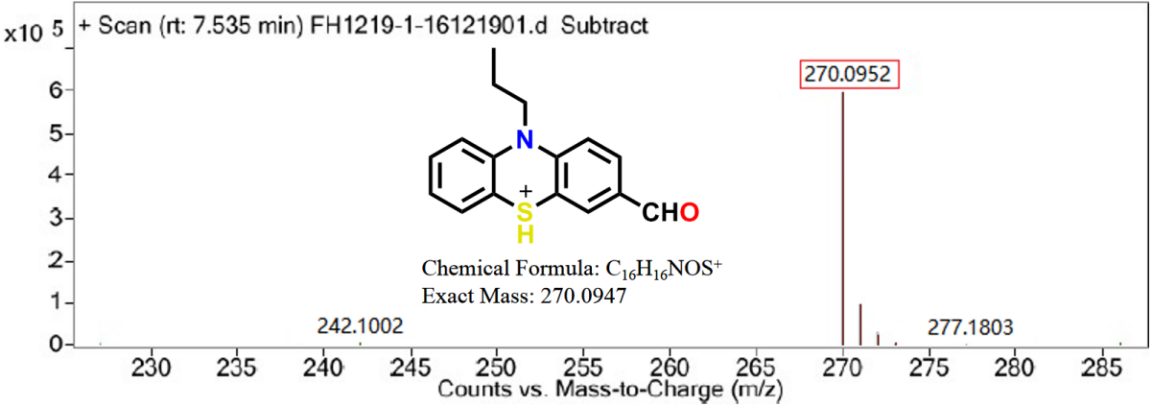


**Fig. S6.** HR-MS of N-propylphenothiazine-3-formaldehyde.


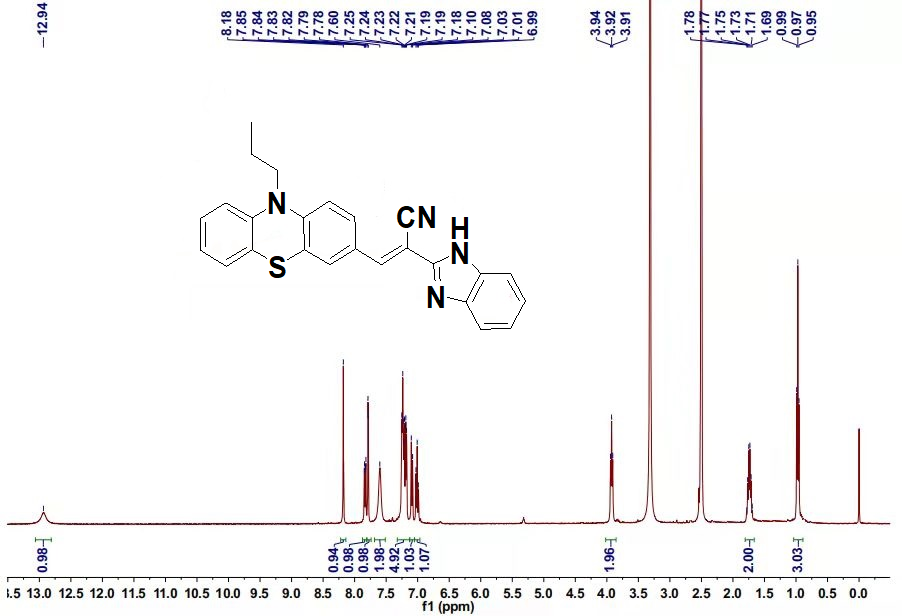


**Fig. S7.** ^1^H NMR of probe **PTA** (DMSO-*d*_6_, 400 MHz).


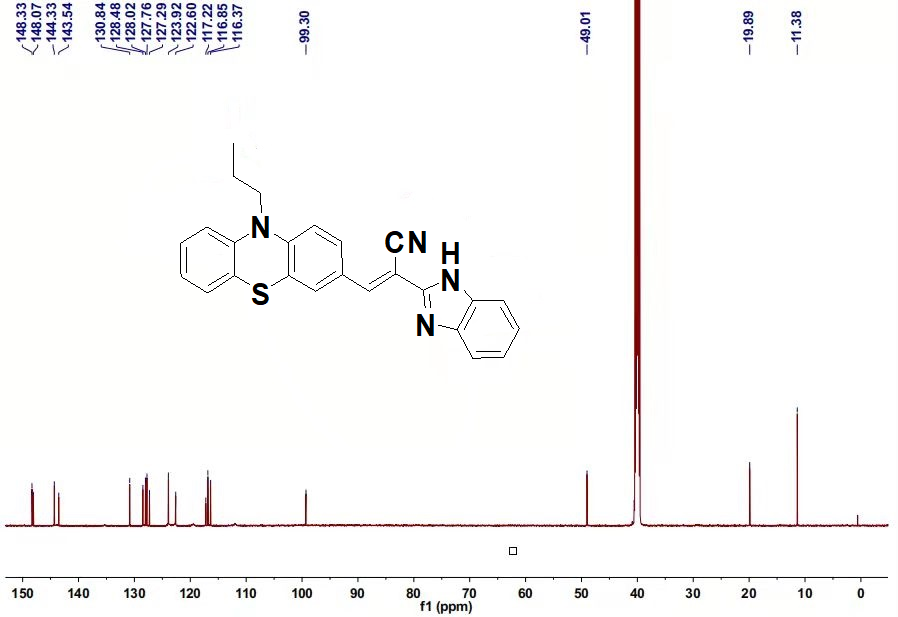


**Fig. S8.** ^13^C NMR of probe **PTA** (DMSO-*d*_6_, 101 MHz).


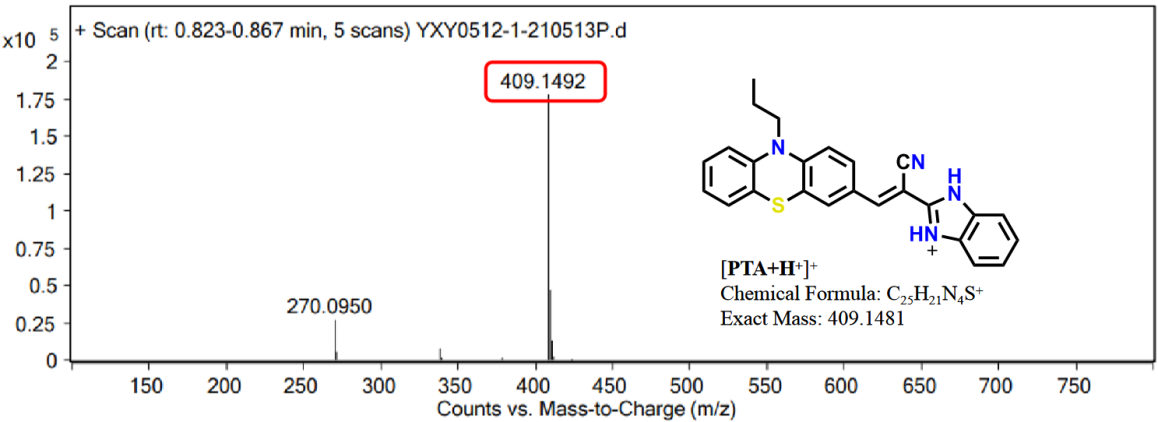


**Fig. S9.** HR-MS of probe **PTA**.


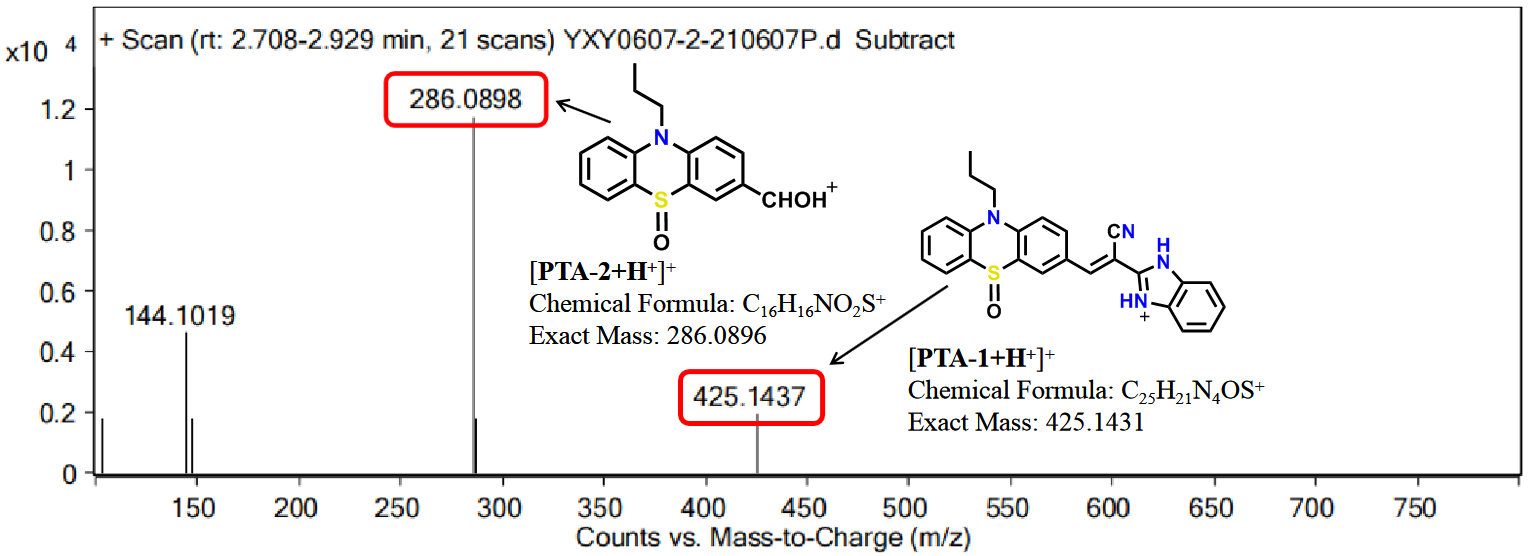


**Fig. S10.** HR-MS of probe **PTA** in the presence of HOCl.

**Theoretical computations**

The ground state structures of **PTA**, **PTA-1** and **PTA-2** were optimized using the density functional theory (DFT) with the Beck’s three-parameter hybrid functional with the Lee-Yang-Parr correlation functional (B3LYP). All atoms were treated with 6-31G(d,p) basis set. All quantum chemistry calculations were performed using the Gaussian 16 package.

**Table S1.** Cartesian coordinates of **PTA** in the ground state (S_0_).

**Coordinates (ground state S_0_)**

-----------------------------------------------------------------------------------------------------------

**Center Atomic Forces (Hartrees/Bohr)**

**Number Number X Y Z**

-----------------------------------------------------------------------------------------------------------

1 6 -0.000004830 -0.000002146 -0.000000725

2 6 -0.000002416 0.000001772 -0.000000501

3 6 0.000002508 -0.000002490 0.000000791

4 6 0.000000732 -0.000001117 0.000000106

5 6 -0.000006233 0.000006289 0.000000886

6 6 0.000006773 -0.000000951 0.000004131

7 16 0.000001477 -0.000000763 0.000004178

8 6 -0.000001548 -0.000005382 -0.000000898

9 6 0.000012932 0.000008994 0.000001134

10 7 -0.000007137 -0.000002371 -0.000007646

11 6 -0.000001769 -0.000002145 -0.000001870

12 6 -0.000001375 0.000005797 0.000001250

13 6 0.000006865 -0.000004185 -0.000000462

14 6 -0.000009553 -0.000000834 0.000002314

15 6 0.000000384 -0.000001323 0.000000872

16 6 -0.000000522 0.000004579 -0.000001143

17 6 0.000000456 -0.000003426 -0.000000902

18 6 0.000002402 -0.000000123 -0.000001580

19 6 0.000003252 -0.000014647 0.000000301

20 6 -0.000010109 0.000001247 0.000000396

21 7 0.000019926 -0.000004439 -0.000000628

22 7 0.000004898 0.000006776 -0.000001236

23 6 -0.000023760 0.000005420 0.000001021

24 6 0.000004646 -0.000003049 -0.000000640

25 6 0.000000337 0.000009784 -0.000000785

26 6 0.000003606 -0.000003223 0.000000077

27 6 -0.000001331 -0.000001836 0.000000181

28 6 0.000001974 0.000000934 -0.000000497

29 6 0.000000199 0.000001443 -0.000000365

30 7 0.000001034 0.000000409 -0.000000615

31 1 0.000000851 0.000000780 0.000000712

32 1 0.000001015 -0.000001841 0.000001555

33 1 -0.000001545 -0.000000065 -0.000000194

34 1 -0.000001112 -0.000001375 -0.000001107

35 1 0.000000437 0.000000005 0.000000437

36 1 -0.000003250 0.000000758 0.000000728

37 1 0.000000524 -0.000000280 -0.000000671

38 1 0.000000600 0.000001624 0.000000826

39 1 -0.000000911 0.000000392 0.000000962

40 1 0.000000210 -0.000000511 0.000000363

41 1 0.000000796 -0.000000775 0.000001177

42 1 -0.000000055 0.000000730 -0.000000390

43 1 0.000000015 0.000000441 -0.000000362

44 1 -0.000000185 0.000000467 -0.000000414

45 1 -0.000000137 0.000000342 0.000000540

46 1 -0.000000611 -0.000000536 -0.000000525

47 1 -0.000000380 0.000000412 0.000000114

48 1 0.000000052 0.000000198 -0.000000033

49 1 -0.000000195 0.000000197 -0.000000329

50 1 0.000000069 0.000000042 -0.000000532

-----------------------------------------------------------------------------------------------------------

**Table S2.** Cartesian coordinates of **PTA-1** in the ground state (S_0_).

**Coordinates (ground state S_0_)**

-----------------------------------------------------------------------------------------------------------

**Center Atomic Forces (Hartrees/Bohr)**

**Number Number X Y Z**

-----------------------------------------------------------------------------------------------------------

1 6 0.000000729 0.000005366 -0.000000984

2 6 0.000003042 -0.000004589 0.000004435

3 6 -0.000003192 -0.000000371 -0.000001955

4 6 0.000001798 0.000004999 0.000001687

5 6 -0.000007678 -0.000009167 0.000000494

6 6 -0.000000337 -0.000001930 -0.000003739

7 16 0.000001590 0.000001545 0.000000358

8 6 0.000001270 -0.000002930 0.000000502

9 6 -0.000008961 0.000002416 0.000000478

10 7 0.000011558 0.000001159 -0.000004291

11 6 0.000000487 0.000001771 -0.000001653

12 6 -0.000000829 -0.000004736 0.000002985

13 6 -0.000001803 0.000003998 -0.000001621

14 6 0.000004048 -0.000000166 -0.000000543

15 6 -0.000002488 0.000003906 0.000002409

16 6 -0.000000161 0.000002306 0.000000990

17 6 0.000001268 0.000000606 0.000000139

18 6 -0.000000786 0.000000061 0.000002322

19 6 0.000005717 0.000000279 -0.000002341

20 6 -0.000004031 -0.000001755 0.000001394

21 7 0.000005528 -0.000001869 -0.000003549

22 7 -0.000004019 0.000001431 0.000000481

23 6 -0.000006460 0.000003049 0.000000594

24 6 0.000005314 -0.000000379 0.000000618

25 6 -0.000005245 0.000000206 0.000000796

26 6 0.000001532 -0.000000465 -0.000000976

27 6 -0.000000454 0.000000333 -0.000000517

28 6 0.000000232 -0.000000471 0.000000007

29 6 -0.000000802 -0.000000372 -0.000000239

30 7 0.000002186 0.000000219 -0.000000532

31 8 -0.000000352 -0.000001006 0.000000637

32 1 -0.000000238 -0.000001650 0.000000701

33 1 -0.000000467 0.000000235 0.000000777

34 1 -0.000000397 0.000000366 0.000000839

35 1 0.000002374 -0.000002431 -0.000001325

36 1 0.000000665 0.000000326 0.000000737

37 1 -0.000000156 -0.000000567 -0.000000290

38 1 -0.000000847 0.000000012 0.000000256

39 1 0.000001046 -0.000000320 0.000000148

40 1 -0.000002859 -0.000002227 -0.000000711

41 1 0.000001587 0.000002300 0.000003111

42 1 0.000000397 0.000000490 -0.000000883

43 1 -0.000000005 -0.000000086 -0.000000859

44 1 -0.000000127 0.000000149 -0.000000709

45 1 0.000000085 -0.000000067 -0.000000401

46 1 -0.000000161 -0.000000634 -0.000000110

47 1 0.000001051 0.000000883 0.000001150

48 1 -0.000000179 -0.000000474 -0.000000477

49 1 -0.000000344 -0.000000263 -0.000000419

50 1 -0.000000257 0.000000141 -0.000000083

51 1 0.000000130 0.000000373 0.000000163

-----------------------------------------------------------------------------------------------------------

**Table S3.** Cartesian coordinates of **PTA-2** in the ground state (S_0_).

**Coordinates (ground state S_0_)**

-----------------------------------------------------------------------------------------------------------

**Center Atomic Forces (Hartrees/Bohr)**

**Number Number X Y Z**

-----------------------------------------------------------------------------------------------------------

1 6 0.000000249 -0.000005369 0.000004271

2 6 -0.000004472 0.000009161 -0.000001867

3 6 0.000009967 -0.000003028 -0.000001358

4 6 -0.000013673 0.000012905 0.000004415

5 6 0.000011670 -0.000012672 -0.000004520

6 6 -0.000010951 0.000004055 0.000000897

7 16 -0.000002886 -0.000009406 0.000000745

8 6 0.000016082 0.000015246 -0.000001571

9 6 -0.000012221 -0.000016695 -0.000000498

10 7 -0.000001410 -0.000006656 0.000003315

11 6 -0.000015812 -0.000004141 0.000000075

12 6 0.000004125 0.000007677 -0.000001279

13 6 -0.000003991 0.000002294 -0.000000501

14 6 0.000017804 0.000004297 0.000005054

15 6 -0.000000062 0.000011570 -0.000006310

16 6 0.000000542 0.000004390 0.000010736

17 6 0.000000082 0.000006255 0.000005956

18 6 0.000001953 -0.000005010 0.000005808

19 8 -0.000001157 0.000000368 -0.000000324

20 8 0.000001272 -0.000004945 0.000001423

21 1 0.000000397 0.000000112 -0.000003048

22 1 0.000000829 -0.000000908 0.000000412

23 1 -0.000001308 0.000000505 -0.000000347

24 1 -0.000001063 -0.000001181 0.000000762

25 1 0.000001621 0.000000540 -0.000000643

26 1 -0.000000143 -0.000001176 -0.000001012

27 1 0.000000994 -0.000001278 0.000000162

28 1 0.000004859 0.000002048 -0.000002308

29 1 -0.000005895 0.000001431 -0.000002413

30 1 -0.000000268 -0.000004913 -0.000005320

31 1 0.000001278 -0.000002783 -0.000005895

32 1 -0.000000621 -0.000000355 -0.000000358

33 1 0.000001179 -0.000000717 -0.000000134

34 1 0.000000040 -0.000002026 -0.000001252

35 1 0.000000991 0.000000406 -0.000003073

-----------------------------------------------------------------------------------------------------------

**Fig. S11.** Fluorescence intensities of probe **PTA** (10 μM) at 550 nm and 680 nm within 45 h in PBS buffer (DMSO:H_2_O=1:9,20 mM,pH=7.4). *λ_ex_*=415 nm.

**Fig. S12.** Curve of the fluorescence intensity ratio of probe **PTA** (10 μM) at 550 nm and 680 nm at different pH (pH = 3-11.5) before and after HOCl was added.

**Fig. S13.** Time-dependent fluorescence response at 680 nm of **PTA** (10 μM) in the presence of 250 μM HOCl in PBS buffer (DMSO:H_2_O=1:9,20 mM,pH=7.4). *λ_ex_*=415 nm.

**Fig. S14.** The viability of A549 cells incubated with **PTA** (0-30 μM) for 24 h.


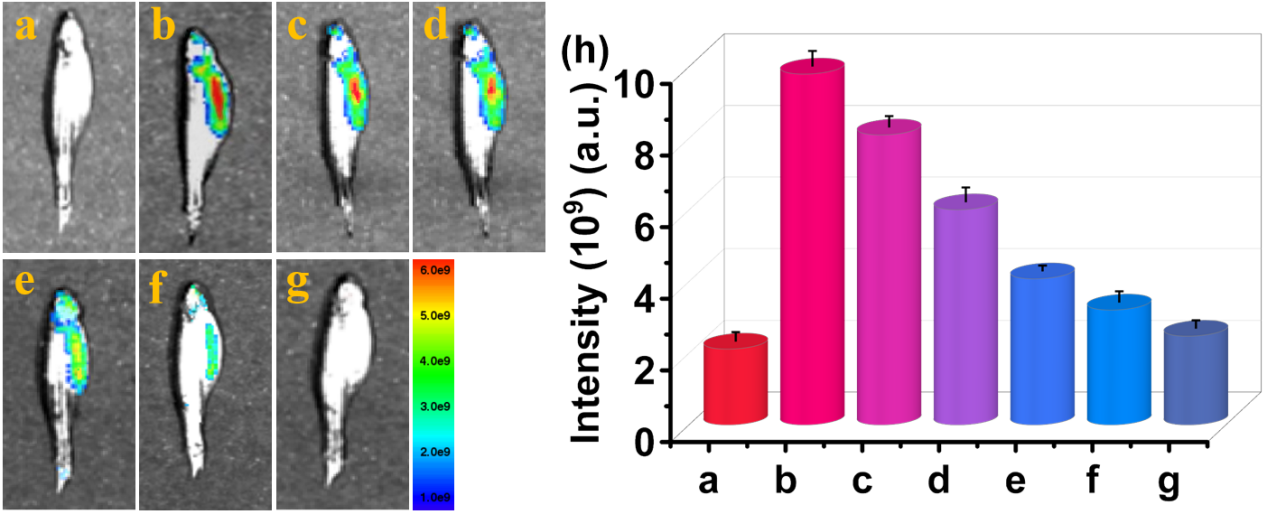


**Fig. S15.** Fluorescence imaging oral-feeding HOCl in zebrafish by using **PTA**. (a) zebrafish only, (b) zebrafish stained with **PTA** (10 μM) for 5 min only, (c) zebrafish pretreated with **PTA** (10 μM) in (b) was put into HOCl solution for (c) 1 min, (d) 2 min, (e)5 min, (f)10 min, (g)15 min, (h) The mean fluorescence intensity of the luminescence regions at different times in (a-g). Zebrafish was imaged using an excitation filter (500 nm) and emission filter (660 nm).


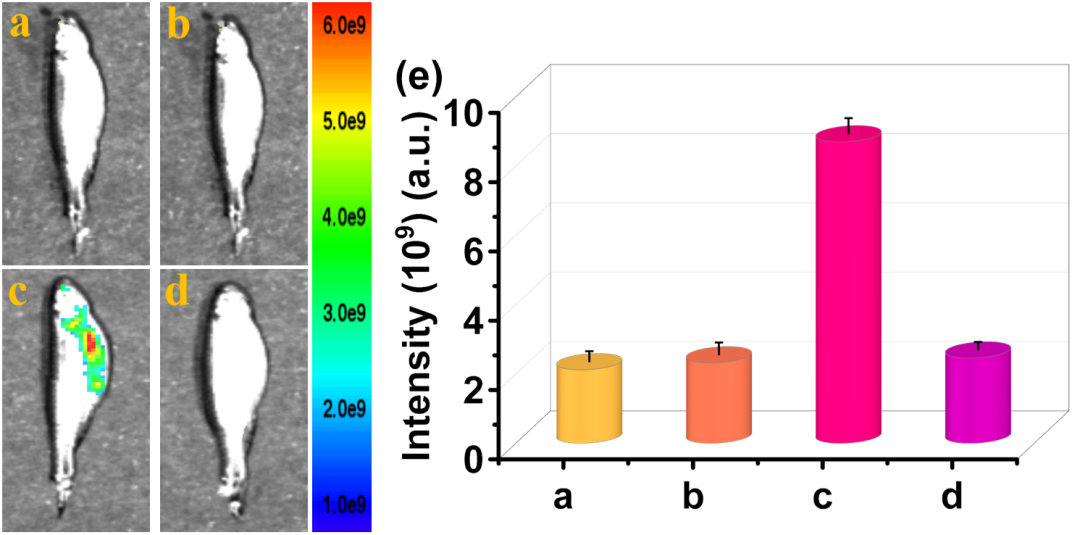


**Fig. S16.** Fluorescence imaging endogenous HOCl production in zebrafish by using **PTA** . (a) zebrafish only, (b) zebrafish was stimulated with LPS (2 μg mL^-1^) for 3 h, (c) zebrafish stained with **PTA** (10 μM) only for 5 min, (d) zebrafish pretreated with LPS (2 μg mL^-1^) for 3 h and then stained with **PTA** (10 μM) for 5 min, (e) The mean fluorescence intensities of the areas of interest at different times shown in (a-d). Zebrafish was imaged using an excitation filter (500 nm) and emission filter (660 nm).

**Table S4**. Comparison of this work with reported fluorescent probes for HOCl detection.

| **Probes** | **Emission wavelength** | **Detection limit** | **Response time** | **Monitoring of HOCl in rheumatoid arthritis model** | **Monitoring of rheumatoid arthritis treatment response** | **Detection of HOCl in live**  **animals** | **“Naked eye” detection of HOCl in real water samples** | **Ref.** |
| --- | --- | --- | --- | --- | --- | --- | --- | --- |
| **Probe-1** | 427 nm | 208.9 nM | 3s | No | No | No | No | 1 |
| **Probe-2** | 575 nm | 17.3 nM | 4 s | Yes | Yes | Zebrafish  mouse | No | 1 |
| **TJM** | 730 nm | 0.11 μM | 7 s | No | No | Zebrafish  mouse | No | 2 |
| **NQ** | 479 nm/551 nm | 21 nM | 20 s | No | No | No | No | 3 |
| **NIR-ClO** | 740 nm | 28.3 nM | 60 s | No | No | mouse | No | 4 |
| **MYQ** | 462 nm/655 nm | 25.3 nM | 6 min | No | No | No | No | 5 |
| **NRH-O** | 710 nm/500 nm | 7.14 nM | 1 min | No | No | No | No | 6 |
| **PTZ-BT** | 642 nm | 0.64 μM | —— | No | No | No | No | 7 |
| **Fcoum-S** | 526 nm/602 nm | 0.15 μM | 10 min | No | No | No | No | 8 |
| **NNH** | 525 nm | 29.3 nM | —— | No | No | No | No | 9 |
| **Naph-DFOB** | 509 nm/628 nm | 15.2 nM | 20 s | No | No | Zebrafish | No | 10 |
| **probe DAB** | 660 nm | 1.46 μM | 1 min | No | No | Zebrafish | No | 11 |
| **BON** | 525 nm | 0.27 μM | 5 min | No | No | No | No | 12 |
| **PTZ-2Cy** | 488 nm/595 nm/600 nm | 0.038 μM | —— | No | No | No | No | 13 |
| **PyOX** | 680 nm | 2.4 nM | 10 s | No | No | mouse | No | 14 |
| **HQ** | 550 nm | 6.5 nM | 25 s | Yes | Yes | Zebrafish  mouse | Yes | 15 |
| **PTA** | 550 nm/680 nm | 33.9 nM | 45 s | Yes | Yes | Zebrafish  mouse | Yes | **This work** |

**References:**

1. H. Feng, Z. Zhang, Q. Meng, H. Jia, Y. Wang, R. Zhang, *Adv. Sci.* **2018**, *8*, 1800397.
2. M. He, M. Ye, B. Li, T. Wu, C. Lu, P. Liu, H. Li, X. Zhou, Y. Wang, T. Liang, H.n Li, C. Li, *Sensor. Actuat. B-Chem.* **2022**, *364*, 131868.
3. C. Zhang, X. Li, Y. Jiang, Y. Zhang, Y. Xie, Y. Sun, C. Liu, *Spectrochim. Acta A* **2022**, *283*, 121736.
4. Q. Luo, Z. Luo, H. Zeng, Y. Xiao, Y. Peng, G. Liu, *Spectrochim. Acta A* **2022**, *273*, 121017.
5. X. Xu, H. Ding, Q. Zhang, G. Liu, S. Pu, *Dyes Pigments* **2022**, *207*, 110776.
6. L. Shangguan, J. Wang, X. Qian, Y. Wu, Y. Liu, *Anal. Chem.* **2022**, *94*, 11881−11888.
7. L. Wang, X. Chen, Q. Xia, R. Liu, J. Qu, *Ind. Eng. Chem. Res.* **2018**, *57*, 7735−7741.
8. M. Cho, V. Nguyen, J. Yoon, *ACS Meas. Sci. Au* **2022**, *2*, 219−223.
9. P. Xing, Y. Feng, Y. Niu, Q. Li, Z. Zhang, L. Dong, C. Wang, *Chem.–A Eur. J.* **2018**, *24*, 5748−5753.
10. S. Han, B. Ding, H. Xiong, B. Zuo, Y. Ye, H. Li, X. Song, *Analyst* **2022**, *147*, 5300–5305.
11. L. Zhen, J. Lan, S. Zhang, L. Liu, R. Zeng, Y. Chen, Y. Ding, *Anal. Methods* **2022**, *14*, 2147–2152.
12. W. Cheng, C. Ren, S. Liu, W. Jiang, X. Zhu, W. Jia, J. Cheng, Z. Liu, *New J. Chem.* **2022**, *46*, 18010–18017.
13. L. Liang, Y. Sun, C. Liu, X. Zeng, J. Zhao, *Dyes Pigments* **2021**, *190*, 109344.
14. L. Wang, J. Liu, H. Zhang, W. Guo, *Sensor. Actuat. B-Chem.* **2021**, *334*, 129602.
15. X. Yang, Y. Wang, Z. Shang, Z. Zhang, H. Chi, Z. Zhang, R. Zhang, Q. Meng, *RSC Adv*. **2021**, *11*, 31656–31662.
